# On Medical Reporting

**Published:** 1829-01-01

**Authors:** 


### MEDICAL REPORTING.

Our readers can testify that we have always advocated the utility and the propriety of *authenticated* reports from public hospitals and from medical societies. It is against the system of anonymous, false, or caricatured reports that we have ever lifted up our voice. The cases occurring in public institutions, are something like the viva voce discussions in medical societies :—they are transitory scenes that are lost to the community at large, excepting so far as they are turned to advantage by those who are immediate witnesses of them. So the discussions in medical societies, are mere words that are lost in air to all except the auditors present. The fixation and preservation of the events in the one case, and of the discussions in the other, contribute to the general good, and deserve to be encouraged. But this encouragement must surely presuppose the most rigid impartiality—the most strict veracity—the most scrupulous correctness. That these last requisites have not always attended the system of anonymous or unauthenticated reporting, needs no proof in the present day; and we have always been of opinion, that the best safe-guard against false or garbled reports would be found in the appointment of accredited, and consequently *responsible* reporters. It has been urged, indeed, that *these* last are not so *independent* as anonymous observers. Most undoubtedly they are not at liberty to distort facts, suppress the truth,—and substitute fictions. The power of doing such things uncontrolled, is the only superior independence which the anonymous reporter possesses over the accredited agent. The latter has nothing to fear from the report of truth—the former from that of fiction. This is the difference. How we have been reviled for maintaining these principles is well known to the profession; yet the Editor of the Lancet is now taking steps to adopt them himself. He has applied to two Medical Societies for permission to have responsible reporters at their meetings, in preference to anonymous agents; and we would strongly advise the said societies to *passively* permit the deputed reporters —that is, to neither sanction nor prohibit their proceedings; but to wink at them so long as the reports are faithfully made, reserving the option of taking such steps as may appear advisable, in case the reports are incorrectly set forth. In the first place, these societies cannot prevent anonymous reporting, even if it were desirable so to do:—in the second place, and as a consequence, it is obviously an object to secure, or at least to encourage that kind of reporting which promises most fidelity and candour. This will be best attained by the reporters being known. This circumstance will be a constant check on misrepresentation—a perpetual stimulus to accuracy.

The Editor of the LANCET is not defective in penetration; and we have no doubt that he begins to see the policy of reverting to those principles of justice, moderation, suavity, and impartiality, which form the most solid and durable basis, after all, of a medical journal. The excitement produced by outrageous satire, ridicule, and party-feeling is too vivid to last long. It must wear out both writers and readers. No man can live long on champagne or imperial tokay, without solid nutriment. Nausea and satiety are sure to succeed. The plainest fare is certain to be the longest relished.

---

